# F4, a collagen XIX-derived peptide, inhibits tumor angiogenesis through αvβ3 and α5β1 integrin interaction

**DOI:** 10.1080/19336918.2021.1951425

**Published:** 2021-07-24

**Authors:** Jean-Baptiste Oudart, Matthieu Villemin, Bertrand Brassart, Christèle Sellier, Christine Terryn, Aurélie Dupont-Deshorgue, Jean Claude Monboisse, François-Xavier Maquart, Laurent Ramont, Sylvie Brassart-Pasco

**Affiliations:** a UMR CNRS/URCA 7369, Matrice Extracellulaire et Dynamique Cellulaire (MEDyC), Université de Reims Champagne Ardenne (URCA), Reims, France; b CHU Reims, Service Biochimie-Pharmacologie-Toxicologie, Reims, France; c PICT, Université de Reims Champagne Ardenne (URCA), Reims, France

**Keywords:** Extracellular matrix, collagen XIX, matrikine, integrin, angiogenesis

## Abstract

We previously demonstrated that F4 peptide (CNPEDCLYPVSHAHQR) from collagen XIX was able to inhibit melanoma cell migration*in vitro* and cancer progression in a mouse melanoma model. The aim of the present work was to study the anti-angiogenic properties of F4 peptide. We demonstrated that F4 peptide inhibited VEGF-induced pseudo-tube formation on Matrigel by endothelial cells and endothelial sprouting in a rat aortic ring assay. By affinity chromatography, we identified αvβ3 and α5β1 integrins as potential receptors for F4 peptide on endothelial cell surface. Using solid phase assays, we proved the direct interaction between F4 and both integrins. Taken together, our results demonstrate that F4 peptide is a potent antitumor agent inhibiting both angiogenesis and tumor cell migration.

## Introduction

During tumor growth, more nutrient and oxygen are required [1]. For that purpose, tumors develop a neo-vascular network [2]. This vascular network favors tumor cell proliferation, invasion and metastatic dissemination [3]. This process, commonly named ‘angiogenic switch’, corresponds to an imbalance between pro- and anti-angiogenic molecules [4,5]. Cells that participate in this process, first of all the endothelial cells [6,7], acquire finely regulated proliferation, migration, and assembly properties, resulting in the generation of new vessels favoring tumor growth [8,9].

Many processes related to angiogenesis are mediated *via* integrins, cell adhesion heterodimeric receptors, composed of α and β subunits [10,11]. Pro-angiogenic factor as vascular growth factors (VEGFs), fibroblast growth factors (FGFs), platelet-derived growth factors (PDGFs) promote tumor angiogenesis [12,13]. Their numerous signaling pathways are interconnected with those triggered by integrins [14].

The extracellular matrix plays a major role in the control of tumor progression and especially tumor angiogenesis [15]. Basement membranes are specialized extracellular matrix, located at the interface between endothelia and connective tissue in blood vessel walls [16]. Basement membrane components interact with cells *via* cell surface receptors, especially integrins, to regulate many biological functions, such as adhesion, differentiation, proliferation, migration. Matrikines are bioactive fragments released by limited proteolysis of extracellular matrix macromolecules [17] involved in many physio-pathological processes such as tumor growth [18,19]. Among them, we and other have previously demonstrated that the different non-collagenous NC1 domains of type IV collagen exhibit anti-tumor and anti-angiogenic activity [20].

Type XIX collagen, that belongs to the FACIT family (Fibril-Associated Collagens with Interrupted Triple helices), is a minor collagen associated to the basement membrane zone [21]. We showed that the NC1(XIX) domain (NPEDCLYPVSHAHQRTGGN), located at the C-terminus of the α1(XIX) collagen chain, strongly inhibits *in vivo* tumor growth as well as angiogenesis in a mouse melanoma model [22,23]. It inhibits melanoma cell migration and invasion. We also showed that plasmin, one predominant enzyme involved in tumor invasion and angiogenesis, cleaves type XIX collagen and releases a bioactive peptide/matrikine. This peptide, that we called F4, reproduced the whole NC1(XIX) domain effect on melanoma cell migration [24].

In the present paper, we studied F4 effects on endothelial cell proliferation and pseudo-tube formation *in vitro* as well as on endothelial cell sprouting in an *ex vivo* model. We also tried to identify F4 receptor(s) on endothelial cell surface.

## Results

### NC1(XIX) and F4 do not affect HUVEC proliferation

**Figure 1.**
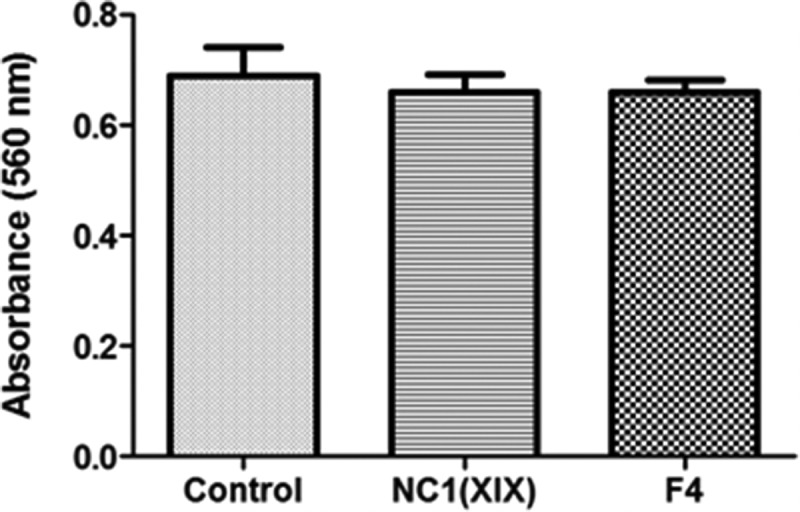
**NC1(XIX) and F4 does not affect endothelial cell proliferation**. 5,000 HUVEC were seeded in a 96 well plate and incubated with or without 40 μM NC1(XIX) or F4 for 48 hours. After washing, cells were fixed with glutaraldehyde and stained with crystal violet. Dye was eluted with 10% acetic acid and absorbance was read at 560 nm. The values are the means ± SD (n = 16). The experiment was repeated three times (N = 3) and the results presented in the figure correspond to the more representative one

The effects of NC1(XIX) or F4 on HUVEC proliferation were assessed using the crystal violet coloration after 48 h of incubation with or without peptides. Cell proliferation was not significantly modified whatever the culture conditions (Figure 1).

### NC1(XIX) and F4 inhibit HUVEC in vitro pseudo-tube formation and ex vivo migration

**Figure 2.**
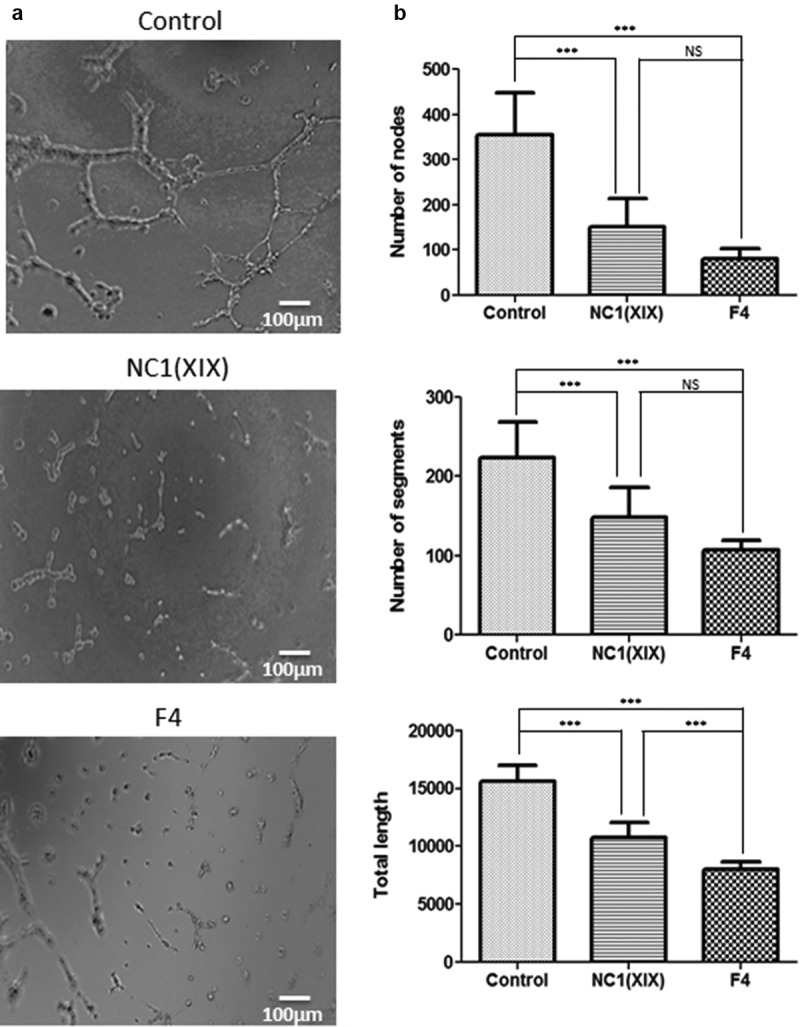
**NC1(XIX) and F4 inhibit endothelial cell *in vitro* pseudotube formation**. 50,000 HUVEC were seeded in Matrigel coated well of a 48 well-plate and incubated with or without 40 μM NC1(XIX) or F4. Pseudotube formation was observed and photographed under an inverted microscope after 8 hours (a). The number of segments, of nodes and the total length of pseudotubes were quantified by the ImageJ software, using the Angiogenesis Analyzer tool (b). The values are the means ± SD (n = 8 replicates). *** *p* < 0.001). The experiment was repeated three times (N = 3)

HUVEC were seeded onto Matrigel and incubated with or without 40 µM NC1(XIX) or F4 for 8 h and capillary network was photographed under an inverted microscope (Figure 2(a)). Both NC1(XIX) and F4 strongly inhibited pseudo-tube formation compared to control. Quantitative evaluation of the number of nodes showed a 57% (p < 0.001) and a 77% (p < 0.001) decrease following NC1(XIX) and F4 treatment, respectively, compared to control (Figure 2(b)). In the same way, they strongly decreased the number of segments (33% (p < 0.001) and 52% (p < 0.001), respectively) and the total length of capillary tubes (31% (p < 0.001) and 49% (p < 0.001), respectively) compared to control (Figure 2(b)). F4 peptide showed a stronger inhibition of the number of nodes, segments and of the total length of capillary network than NC1(XIX) peptide (Figure 2(b)).

**Figure 3.**
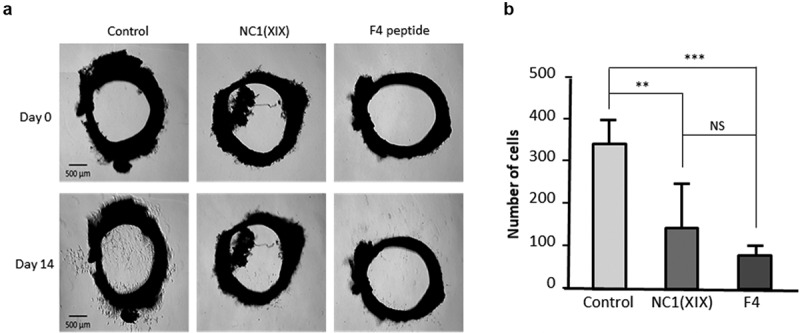
**NC1(XIX) and F4 inhibit endothelial cell *ex vivo* migration**. Rat aortic rings were embedded in Matrigel in 24 well-plate and then incubated with or without 40 μM NC1(XIX) or F4 for 14 days. Micro-vessel outgrowth was observed under an inverted microscope after 14 days and photographed (a). Cell outgrowth from the tissue explant was measured using a home-made macro based on ImageJ analysis software. The results are presented as mean ± SD (n = 5 replicates) (b). The experiment was repeated twice (N = 2)

Moreover, anti-angiogenic effects of NC1(XIX) and F4 peptide were then investigated *ex vivo* in the aortic ring assay. Sprague Dawley rat aortic rings were embedded into Matrigel and incubated with or without 40 μM NC1(XIX) or F4. After 14 days, capillary outgrowth from aortic ring was photographed under an inverted microscope (Figure 3(a)). NC1(XIX) and F4 significantly decreased cell outgrowth compared to control (−57% (p < 0.01) and −78% (p < 0.001), respectively) (Figure 3(b)). F4 effect was even stronger than NC1(XIX) effect.

### Identification of αvβ3 and α5β1 integrins as F4 potential receptors on HUVEC

**Figure 4.**
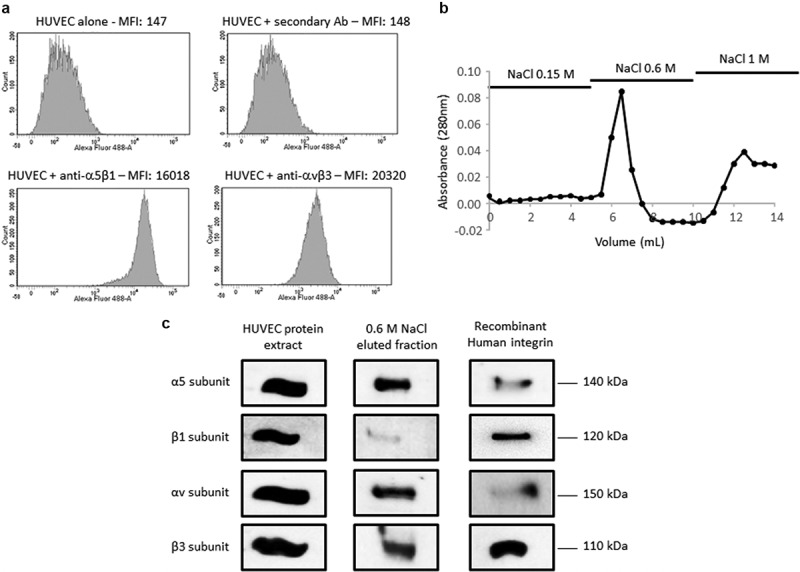
**F4 binds to αvβ3 and α5β1 integrins on HUVEC**. (a) HUVECs were analyzed by flow cytometry for the expression of αvβ3 and α5β1 integrins. (b) HUVEC extracts were submitted to F4 affinity chromatography. Bound proteins were eluted with increasing concentrations of NaCl (0.15, 0.6 and 1 M) and the elution profile was checked by recording the absorbance at 280 nm. (c) Eluted samples were then analyzed by SDS-PAGE and western blot HUVEC total extracts and recombinant αvβ3 and α5β1 integrins were used as positive controls. The 0.6 M eluted sample revealed bands which matched the molecular weight of the α5, β1, αv and β3 recombinant integrin subunits. The experiment was repeated twice (N = 2)

As several ECM macromolecules were reported to mediate endothelial cell migration in a αvβ3 and α5β1 integrin-dependent manner [11], we checked for their expression in the pooled HUVEC we used in our experiment using flow cytometry. Both integrins were expressed on HUVEC surface (Figure 4(a)). Affinity chromatography was used to identify F4 receptor(s) on HUVEC. Protein extracts were loaded onto a F4 peptide-Sepharose column. Bound proteins were eluted with increasing concentrations of NaCl (0.15, 0.6 and 1.0 M) and the elution profile was followed by measuring protein absorbance at 280 nm (Figure 4(b)). Eluted samples were submitted to SDS-PAGE and analyzed by western blot using anti-αv, β3, α5 and β1 subunit antibodies (Figure 3(c)). Total HUVEC protein extract and recombinant integrins were used as positive controls. The anti-α5 and -β1 subunit antibodies revealed two bands in 0.6 M NaCl eluted sample which matched the 140 and 120 kDa bands of the recombinant α5β1 integrin, respectively. In the same way, the anti-αv and -β3 subunit antibodies revealed two bands which matched the 150 and 110 kDa bands of the recombinant αvβ3 integrin, respectively. Taken together, these results suggested a binding of F4 on α5β1 and αvβ3 integrins.

### F4 peptide directly binds to αvβ3 and α5β1 integrins

**Figure 5.**
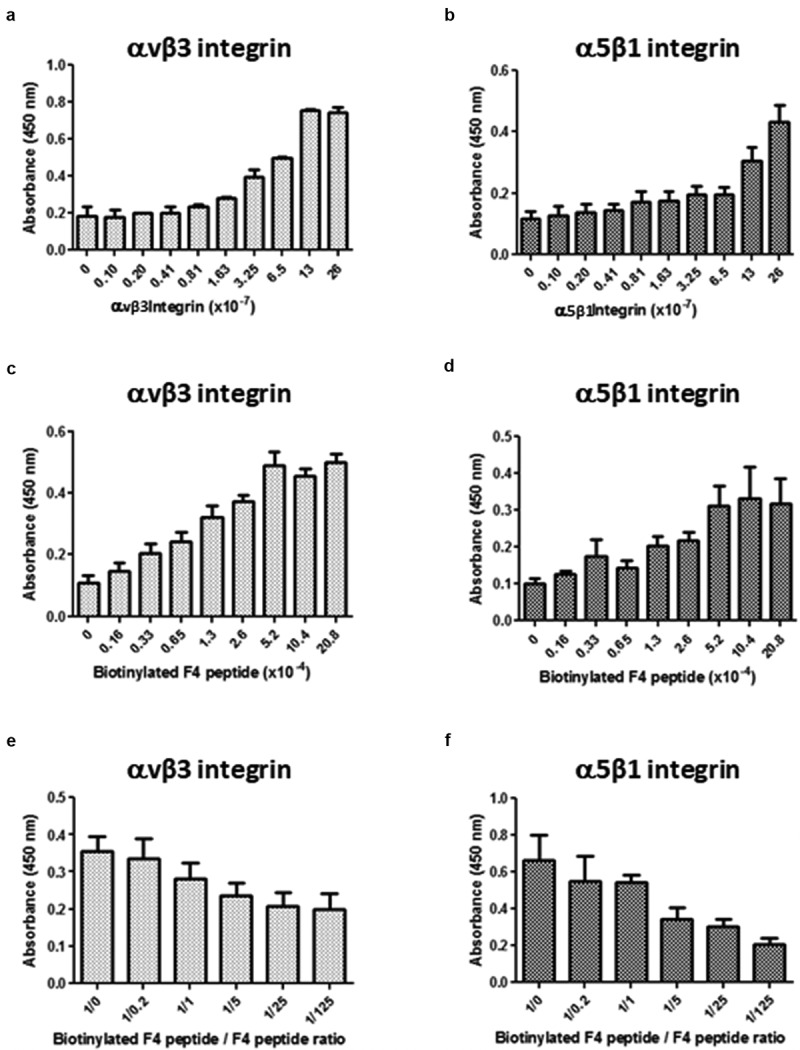
**F4 directly binds to αvβ3 and α5β1 integrins**. Direct interaction between αvβ3 or α5β1 integrin and F4 was studied using solid phase assays as described in the material and method section. The F4 peptide was biotinylated to allow its detection using the streptavidin-peroxidase complex. Different conditions were used. (a-b) The amounts of coated αvβ3 (a) or α5β1 integrin (b) were increased while biotinylated F4 peptide was kept constant. (c-d) The concentration of biotinylated F4 peptide was increased while coated amounts of αvβ3 (c) or α5β1 (d) integrins were kept constant. (e-f) Competitive assays were performed using increasing concentrations of F4 peptide while biotinylated-F4 peptide and αvβ3 (e) or α5β1 (f) integrins were kept constant. The solid phase assays were repeated twice (N = 2; n = 2)

A putative direct interaction of F4 with αvβ3 or α5β1 integrin was investigated using solid phase assays as described in the material and method section. The F4 peptide was biotinylated to allow its detection using the streptavidin-peroxidase complex. Bindings of biotinylated-F4 on αvβ3 or on α5β1 were dependent both on the receptor (Figure 5(a,b)) and on ligand (Figure 5(c,d)) concentrations. Moreover, competitive assays were performed to confirm the specificity of the interaction. Increasing amounts of unlabeled F4 peptide were added to the biotinylated-F4 peptide solution. Biotinylated-F4 peptide binding to αvβ3 (Figure 5(e)) or α5β1 (Figure 5(f)) decreased consequently. Taken together, the results confirm that F4 peptide binds to both αvβ3 and α5β1 integrins in a direct manner.

## Discussion

ECM macromolecules determine tissue physical and mechanical properties, but they also influence cell behavior in many physio-pathological processes, among which tumor angiogenesis [25]. Proteolytically derived fragments from NC1 domain of type IV, XV and XVIII basement membrane collagens were reported to inhibit tumor angiogenesis [26]. We previously demonstrated that plasmin releases a F4 fragment from the type XIX collagen NC1 domain [24]. In the present paper, we prove that the F4 matrikine exerts anti-angiogenic effects on *in vitro* pseudo-tube formation and on *ex vivo* aortic endothelial sprouting even greater than the whole NC1(XIX) domain. Comparisons between NC1(XIX) and F4 structures were performed by molecular modeling [24]. In the F4 fragment, unlike fragment NC1(XIX), the presence of a disulfide bridge between two cysteine residues promotes the formation of a natural loop that might stabilize its 3D-structure and potentiates its anti-angiogenic activity compared to NC1(XIX) domain.

By covalently coupling NC1(XIX) to agarose beads, Chen *et al*. [27] were able to purify α5β1 integrin from cortical protein extract. They demonstrated that NC1 (XIX) was involved in triggering inhibitory nerve terminal assembly *in vitro*. RGD peptides blocked this inhibitory effect.

Many integrins, including αvβ3 and α5β1, recognize the tripeptide Arg–Gly–Asp (RGD) in their ligands. Fibronectin and vitronectin favor tumor angiogenesis through α5β1 and αvβ3, respectively [11]. Both integrins are up-regulated on endothelial cells within malignant tumors and represent target of choice for angiogenesis blockade. The F4 peptide binds both to αvβ3 and α5β1 integrins and may be used as a potent anti-angiogenic agent by competing with αvβ3 and α5β1 ligands or counteract their effects.

Anti-angiogenic drugs are commonly used in daily practice, especially in the treatment of a wide number of cancers. For example, bevacizumab (a monoclonal anti-VEGF antibody) is used in combination with conventional chemotherapy, targeted therapy or immunotherapy [28]. However, development of resistance mechanisms occurs in several tumor types through angiogenic ‘escape’ pathways [29,30]. This contributes to tumor escape and may induce tumor progression, enhancement of invasion, and metastasis. Moreover, a wide range of side effects frequently alter the tolerance of this therapy. The development of anti-angiogenic peptides rather than monoclonal antibodies may be useful in this context. Therapeutic peptides present several advantages over proteins or antibodies. They are small in size, diffusible and easy to synthesize. They are generally selective and efficacious signaling molecules that bind to specific cell surface receptors. They are less immunogenic than recombinant antibodies or proteins and well tolerated. They do not accumulate in specific organs (e.g. kidney or liver) and, by the way, have fewer side effects [31,32]. However, their *in vivo* stability might be problematic. The F4 peptide is a matrikine naturally released *in vivo* and its administration to the patient should produce little if any side effects. Moreover, the presence of the intra-chain disulfide bond that spontaneously forms in solution stabilizes the F4 peptide 3D structure and may reinforce its resistance to *in vivo* proteolytic degradation, and therefore increase its biological activity. This was previously reported for proteins such as β-lactamase and Serine protease inhibitors of the Kunitz-bovine pancreatic trypsin inhibitor (BPTI) [33,34] and for peptides such as alpha-CTX MII [35].

In addition to its anti-angiogenic properties, we previously demonstrated that F4 also inhibits *in vitro* tumor cell migration [36] . In conclusion, the anti-migratory and anti-angiogenic properties of F4 peptide make it a promising therapeutic agent.

## Materials and methods

### Peptide synthesis

All peptides were purchased from Proteogenix. The NC1(XIX) peptide sequence was NPEDCLYPVSHAHQRTGGN and F4 peptide sequence was CNPEDCLYPVSHAHQR. The peptides were obtained by solid-phase synthesis using a FMOC (N-(9-fluorenyl) methoxy-carbonyl) derivative procedure. They were then purified by reverse phase high performance liquid chromatography using a C18 column, eluted by a gradient of acetonitrile in trifluoroacetic acid, and lyophilized [37]. Their purity was >95% as assessed by mass spectrometry.

### Cell culture

Human Umbilical Vein Endothelial Cells (HUVEC) (Ref C-12253) were purchased from Promocell. They were grown in Endothelial Cell Growth Medium (ECGM) (Promocell, Ref C-22110: Basal medium supplemented with 0.02 mL/mL Fetal Calf Serum, 0.004 mL/mL Endothelial Cell Growth Supplement, 0.1 ng/mL Epidermal Growth Factor (recombinant human), 1 ng/mL Basic Fibroblast Growth Factor (recombinant human), 90 μg/mL Heparin, 1 μg/mL Hydrocortisone) at +37°C in a humid atmosphere containing 5% CO_2_. PromoCell Detach Kit was used for the detachment of HUVEC according to the manufacturer’s protocols (Promocell, ref C-41200).

### Proliferation assay

For cell proliferation measurement, 5,000 HUVEC were seeded in 96-well plates and cultivated in ECGM with or without 40 μM NC1(XIX) or F4 at +37°C in a humid atmosphere containing 5% CO_2._ After 48 h, cells were washed with PBS, fixed with 1.1% (v/v) glutaraldehyde for 20 min and stained with 0.1% (m/v) crystal violet in HEPES (0.2 M, pH 6) for 20 min. Dye was eluted with 10% (v/v) acetic acid and absorbance was read at 560 nm.

### Capillary tube formation on Matrigel

The ability of HUVEC to form pseudo-tubes was investigated on Matrigel (Corning, ref 356237). Matrigel was loaded into 48-well plate and allowed to polymerize at +37°C for 2 h. 50,000 HUVEC suspended in Basal media supplemented with 0.004 mL/mL Endothelial Cell Growth Supplement, 90 μg/mL Heparin, 1 μg/mL Hydrocortisone with or without 40 μM NC1(XIX) or F4 were seeded into each well. Plates were incubated at +37°C in a humid atmosphere containing 5% CO_2_, 95% for 8 h. The formation of the capillary network was then imaged under an inverted light microscope. Quantitative evaluation of the capillary network was performed using the angiogenesis analyzer tool of ImageJ analysis software. The number of nodes, of segments and the total length of the capillary network were determined.

### Ex vivo aortic ring assay

The *ex vivo* rat aortic ring assay was adapted from Baker *et al*. [38]. Thoracic aorta was dissected from Sprague Dawley rat and stored in Opti-MEM (ThermoFisher, ref 31985062) supplemented with 1% penicillin/streptomycin in a Petri dish. Aorta was then cut into 0.5 mm rings that were incubated in Opti-MEM overnight at +37°C in a Petri dish. Matrigel (7 mg/mL) was loaded in 24-well plate and allowed to polymerize at +37°C for 30 min. The rings were carefully transferred from the Petri dish onto the Matrigel coated wells and were completely embedded by addition of Matrigel. The plate was incubated at +37°C for 30 min. Opti-MEM supplemented with 30 ng/mL VEGF with or without 40 μM NC1(XIX) or F4 was added. Plate was incubated at +37°C in a humid atmosphere containing 5% CO_2_ for 14 days. The supplemented culture medium was changed every 3 days. Rings were imaged under an inverted light microscope at 14 days. Quantitative evaluation of the angiogenic sprouting was determined by measuring the number of endothelial cell outgrowth from the primary tissue explant using a homemade macro based on ImageJ analysis software. The first step consisted in the semi-automatic selection of aortic ring shape at day 0 by manual threshold allowing the design of a top hat filter to obtain a relevant mask of this structure. The mask was then substracted from day 14 image to keep only outgrowing cells. The second step consisted in a manual threshold and the use of ‘Analyze Particules’ tool involving size criteria. The total cell number of cell outgrowing from the ring was determined.

### Flow cytometry

Cells were detached with 50 mM HEPES, 125 mM NaCl, 5 mM KCl and 1 mM EDTA and washed three times with ECGM. They were incubated for 30 min with the first monoclonal antibody (anti-α5β1 ref. 555614 from BD Pharmingen or anti-αvβ3 ref. MAB 1976 from Chemicon; 1 µg/0.5.10^6^ cells) and washed three times with ECGM. They were incubated for 30 min with a goat anti-mouse secondary antibody Alexa Fluor® 488 conjugate (ref. A28175 from Thermo Fisher Scientific; 0.4 µg/0.5.10^6^ cells) and washed three times with ECGM. Then, they were analyzed using a FACS Fortessa flow cytometer (BD Biosciences). Ten thousand cells, gated for forward scatter vs. side scatter, were collected for each sample. Appropriate control isotypes were used. The results were analyzed using Flow Jo software (FlowJo Enterprise). Fluorescence quantification was performed using the median fluorescence intensity (MFI).

### Affinity chromatography

HUVEC were cultured as previously described in a Nunclon® 150 cm^2^ flask. At 80%-confluence, cells were washed with HBSS and scrapped in lysis buffer (RIPA buffer, Sigma, ref R0278-50ML) supplemented with 1% (v/v) Halt Protease Inhibitor Cocktail (ThermoFisher, ref 78430). Cell lysates were incubated for 30 min at +4°C and vortexed every 5 min. Unsoluble debris were pelleted by centrifugation at 10,000 g for 10 min at +4°C. Bio-Rad Protein Assay (Bio-Rad, Marne-La-Coquette, France) was used to determine the protein concentrations according to the manufacturer’s instructions. HiTrap NHS-activated Sepharose High Performance column (GE Healthcare, ref 17–0716-01) was functionalized with F4 peptide previously reduced with 50 mM DTT for 30 min at +37°C. HUVEC protein extracts were chromatographed at +4°C. Unbound protein were removed with 30 mL of washing buffer (10 mM Tris, 1 mM CaCl_2_, 1 mM MgCl_2_, pH 7.6, 1% (v/v) Protease Inhibitor Cocktail and 0.1% (w/v) octylglucoside (Sigma, ref O8001-100 MG)) according to the manufacturer’s instructions. Proteins bound to the affinity column were then eluted with elution buffer (10 mM Tris, pH 7.6, 1% (w/v) PIC and 0.1% (w/v) octylglucoside) supplemented with increasing concentrations of NaCl (0.15, 0.6 and 1 M). Absorbance was read at 280 nm to check protein elution and fractions corresponding to the different NaCl concentrations were pooled and concentrated using NanoSep Centrifugal Devices (cutoff 10 kDa; Pall). SDS sample buffer supplemented with 10 mM DTT was added to the concentrated samples that were then incubated for 30 min at +37°C, denatured for 5 min at +95°C and then submitted to western blotting analysis using anti-integrin subunit antibodies (Table 1). HUVEC extracts and recombinant human αvβ3 and α5β1 integrins (3230-A5-050 and 3050-AV-050, R&D systems) were used as positive controls.

**Table 1.** List of the antibodies and dilutions used in the western blot experiment

Antibody	Reference	Manufacturer	Dilution
Integrin subunit α5	4705	Cell Signaling technology	1/1000
Integrin subunit αv	4711S	Cell Signaling technology	1/1000
Integrin subunit β1	Ab1952P	Millipore	1/1000
Integrin subunit β3	Sc-7312	Santa Cruz Biotechnology	1/1000

### Western blot

20 µg of proteins were solubilized in sample buffer containing 10 mM DTT, reduced for 30 min at +37°C, denatured for 5 min at +95°C and electrophoresed in a 0.1% SDS, 10% polyacrylamide gel. They were then transferred onto an Immobilon-P membrane (Millipore, ref IPVH00010). Membrane was blocked with 5% nonfat dry milk, 0.1% Tween 20 in TBS for 2 h at room temperature, incubated overnight at +4°C with anti-integrin antibody diluted in 1% nonfat dry milk, 0.1% Tween-20 in TBS (Table 1). The membrane was then washed three times with TBS containing 0.1% Tween-20 (TBS-T) and incubated for 1 h at room temperature with a second peroxidase-conjugated anti-IgG antibody. Immune complexes were visualized with the ECL chemiluminescence detection kit (GE Healthcare, ref 28980926) according to the manufacturer’s instructions.

### Solid phase assay for studying ECM protein-integrin interactions

Wells of a 96-well plate were coated with α5β1 (ref. 3230-A5-050, R&D systems) and/or αvβ3 integrin (ref. 3050-AV-050, R&D systems) at different concentrations ranging from 26 × 10^−7^ mol/L to 0.1 × 10^−7^ mol/L (100 μL per well in PBS). The 96-well plate was left overnight at room temperature. The coating was then blocked with TBS containing 5% nonfat dry milk, 1 mM MgCl_2_ and 1 mM CaCl_2_ for 2 h at room temperature. After three washes with washing buffer (0.1% nonfat dry milk, 1 mM MgCl_2_ and 1 mM CaCl_2_ in TBS), wells were filled with 100 μL of a solution containing 26 × 10^−5^ mol/L biotinylated-F4 peptide in washing buffer and the plate was incubated for 90 min at room temperature. After three washes with washing buffer, 100 μL of streptavidin – peroxidase diluted 1/20,000 in washing buffer were added to each well and the plate was incubated for 15 min at room temperature. After four washes with washing buffer, 100 μL per well of tetramethylbenzidine (TMB) (Sigma, ref T0440-100 mL), a peroxidase substrate, were added and the plate was incubated in the dark for 15 min. The enzymatic reaction was stopped by the addition of 50 μL per well of 0.5 M H_2_SO_4_. The intensity of the yellow coloration was measured at 450 nm with an LB940 spectrophotometer (Berthold Technologies).

The optimum integrin amount for the coating was determined using the above protocol and the optimum ligand concentration was sought using different dilutions of the biotinylated-F4 peptide ranging from 20.8 × 10^−4^ to 0.16 × 10^−4^ mol/L.

Following optimum integrin and peptide amount determination, competition experiments were carried out. Wells of a 96-well plate were coated with 26 × 10^−7^ mol/L α5β1 or αvβ3 integrin and nonspecific binding sites were blocked as described above. The wells were then incubated with biotinylated-F4 peptide with or without F4 unbiotinylated competitive peptide (molar ratio ranging from 1/0 to 1/125) diluted in washing buffer and the plate was incubated for 90 min at room temperature. The following steps are the same as described above.

### Statistical analysis

Statistical analysis was performed using an ANOVA test. Results were expressed as mean±SD.
